# m5C-modified circRREB1 promotes lung cancer progression by inducing mitophagy

**DOI:** 10.1186/s13046-025-03460-1

**Published:** 2025-07-14

**Authors:** Dunyu Cai, Xingcai Chen, Haotian Xu, Qingyun Zhao, Xiaodong Zhou, Jiaxi Wu, Shengyi Yuan, Yihong Gao, Deqing Li, Ruirui Zhang, Wenyi Peng, Gang Li, Aruo Nan

**Affiliations:** 1 School of Public Health, Guangxi Medical University, Nanning, 530021 China; 2 Guangxi Key Laboratory of Environment and Health Research, Guangxi Medical University, Nanning, 530021 China

**Keywords:** Lung cancer, circRREB1, m5C modification, Mitophagy

## Abstract

**Background:**

Lung cancer is the most common malignant tumour and the leading cause of cancer-related death. circular RNAs (circRNAs) have important biological functions and are closely related to tumour development. The 5-methylcytosine (m5C) modification can regulate the molecular fate of RNA molecules and thus influence disease development.

**Methods:**

High-throughput RNA sequencing was used to construct the differential expression profiles of circRNAs. The m5C modification of circRREB1 was explored through methylated RNA immunoprecipitation (MeRIP) and crosslinking-immunoprecipitation (CLIP). RNA stability experiments, fluorescence in situ hybridization (FISH), and nuclear-cytoplasmic fractionation experiments were performed to explore the effects of the m5C modification on circRREB1. A system for the silencing and overexpression of circRREB1 was established, and in vitro and in vivo experiments were conducted to study the biological functions of circRREB1. Tagged RNA affinity purification (TRAP), RNA immunoprecipitation (RIP), and coimmunoprecipitation (Co-IP) experiments were conducted to reveal the molecular mechanisms of circRREB1.

**Results:**

In this study, we found that circRREB1 is highly expressed in lung cancer tissues and cells and that patients with high circRREB1 expression have a poor prognosis. We discovered that circRREB1 undergoes the m5C modification mediated by the methyltransferase NSUN2. This modification facilitates its nuclear export via the m5C reader ALYREF. Functional studies demonstrated that circRREB1 promotes lung cancer progression both in vitro and in vivo. Mechanistically, circRREB1 directly binds to HSPA8 and stabilizes it by inhibiting ubiquitin-dependent degradation, thereby inducing mitophagy through the HSPA8/PINK1/Parkin signalling axis and ultimately promoting the development of lung cancer.

**Conclusions:**

This study revealed the presence of m5C modifications on circRREB1 and showed that m5C-modified circRREB1 can induce mitophagy, ultimately promoting lung cancer. These findings provide not only a theoretical basis for further exploration of the mechanisms underlying lung cancer development but also potential targets for lung cancer therapy.

**Graphical Abstract:**

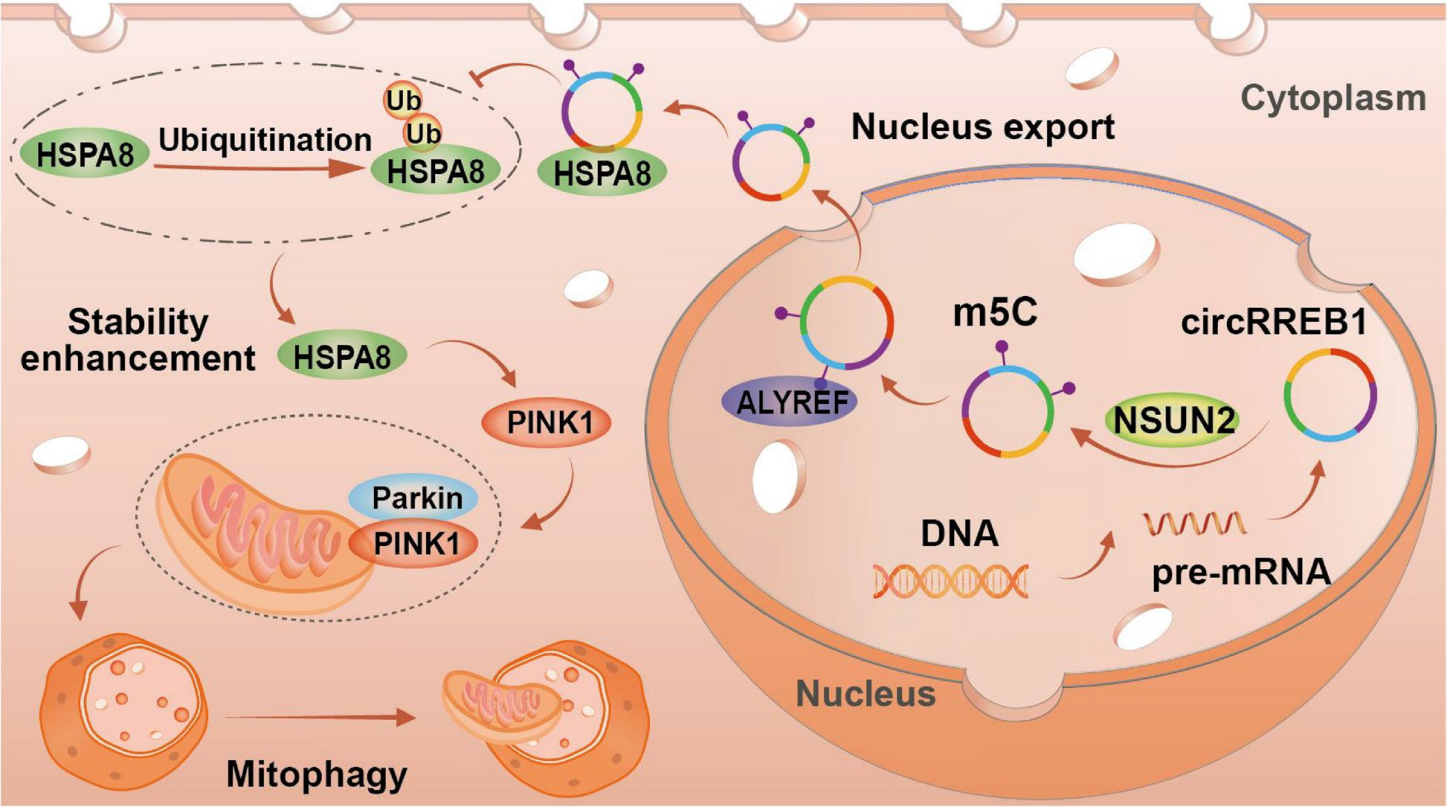

**Supplementary Information:**

The online version contains supplementary material available at 10.1186/s13046-025-03460-1.

## Introduction

Lung cancer is the most common malignant tumour and the leading cause of cancer-related death [1, 2]. Most patients are diagnosed with lung cancer at an advanced stage, which leads to a low five-year survival rate and indicates its serious threat to human health [3]. An in-depth understanding of the molecular mechanisms of lung cancer is crucial for its prevention and treatment. The development of lung cancer is a complex process involving a variety of genetic and environmental factors. In recent years, studies have shown that epigenetic changes are closely related to the development of lung cancer [4]. Recently, epigenetic research has focused mainly on DNA methylation and histone modifications. However, with advancements in genomics and transcriptomics, the role of circRNAs in the development of lung cancer has gradually gained increasing attention.

circRNAs are a class of single-stranded, closed-loop RNA molecules characterized by high stability and strong tissue specificity [5]. Studies have shown that circRNAs can competitively bind to miRNAs, inhibiting the effects of miRNAs on downstream target genes [6]; circRNAs can also bind to RNA-binding proteins (RBPs) to regulate the expression of target genes and can be translated into peptides to regulate the processes of tumorigenesis and tumour progression [7]. Through these functions, circRNAs can participate in the pathophysiological processes of cancer. In lung squamous cell carcinoma, circTP63 competitively binds to miR-873-3p to upregulate FOXM1, promoting the proliferation of lung squamous cell carcinoma [8]; circSCAP interacts with the SF3A3 protein to inhibit the malignant progression of non-small cell lung cancer [9]. However, the mechanisms by which most circRNAs function in cancer remain unknown. Further research on the mechanisms of action of circRNAs in lung cancer will not only clarify the process of lung cancer development but also provide new ideas for lung cancer diagnosis and treatment.

In lung cancer, circRNAs are frequently abnormally expressed. Exploring the underlying reasons for this dysregulation may reveal key aspects of the lung cancer aetiology. m5C methylation is a critical regulator of the RNA molecular fate [10]. While m5C is a well-established modification across various RNA species and plays crucial regulatory roles [11, 12], its specific functions and mechanisms in circRNA biology remain significantly underexplored. The regulatory influence of m5C modification on RNA fate is largely determined by m5C readers [13], which critically regulate RNA export, stability, and translation initiation [14–16]. However, the specific reader responsible for recognizing m5C modifications on circRNAs and governing their downstream regulatory consequences remains unidentified.​ Therefore, ​​identifying the m5C reader for circRNAs and elucidating its role in governing circRNA fate and function during lung cancer tumorigenesis and progression represent a critical, unexplored research frontier.​​ ​​Elucidating this novel m5C-reader-circRNA axis could provide fundamental insights into the molecular mechanisms driving lung cancer development and uncover promising therapeutic targets.​

As research progresses, the pivotal roles of circRNAs in various cellular biological processes have gradually been revealed. circRNAs can influence cancer progression by regulating the level of cellular autophagy [17]. Autophagy is a cellular process that regulates lysosomal processes to control malfunctioning organelles and biomacromolecules [18]. Notably, mitophagy, a type of selective autophagy, can maintain the integrity of intracellular mitochondrial function and cellular homeostasis by identifying and removing dysfunctional mitochondria. An impairment of mitophagy can lead to mitochondrial dysfunction, which in turn affects the occurrence and development of various diseases [19]. PINK1/Parkin-mediated mitophagy is a classic mitophagy pathway. PINK1 is localized to damaged mitochondria, activates Parkin, and marks damaged mitochondria through ubiquitination, supporting the ability of the autophagosome to recognize and remove these dysfunctional mitochondria and thereby maintaining the stability of cellular function [20, 21]. The role of mitophagy in tumorigenesis and the development of cancer is very complex. On the one hand, mitophagy can clear damaged mitochondria, allowing tumour cells to survive under adverse conditions. On the other hand, mitophagy can reduce mitochondrial damage, maintain normal cellular metabolism, reduce the occurrence of cellular oxidative stress, and promote the malignant transformation of cells [22]. Hence, a deeper understanding of the specific role of mitophagy in different types and stages of cancer is crucial for developing effective treatment strategies.

In this study, we identified a circRNA, circRREB1, and found that it is highly expressed in lung cancer. We subsequently revealed that circRREB1 undergoes the m5C modification and is exported from the nucleus in an m5C-dependent manner via ALYREF. In terms of its biological function, we clarified that circRREB1 promotes the development of lung cancer both in vitro and in vivo. Mechanistically, circRREB1 binds to HSPA8, which in turn increases the expression of PINK1, promotes mitophagy and ultimately promotes the development of lung cancer. In summary, our research suggests that circRREB1 may be a key oncogenic factor, providing new ideas for the clinical diagnosis and treatment of lung cancer.

## Materials and methods

### Cell culture

In this study, lung cancer cell lines (A549, H1299, H460 and HCC827 [RRID:CVCL_0023, RRID:CVCL_0060, RRID:CVCL_0459 and RRID:CVCL_2063]) and human bronchial epithelial cells (BEAS-2B [RRID:CVCL_0168]) were purchased from the American Type Culture Collection (ATCC); 293 T human embryonic kidney cells (HEK-293 T [RRID:CVCL_0063]) were purchased from Guangzhou Cellcook Biotech Co., Ltd. (Guangzhou, China). BEAS-2B cells were cultured in BEGM (Lonza, CC-3171) medium; HEK-293 T cells were cultured in DMEM (Servicebio, G4511) supplemented with 10% foetal bovine serum (Gibco, 10099141 C) and a 1% penicillin-streptomycin solution (Servicebio, G4003); A549 cells were cultured in Ham’s F-12 K medium (Servicebio, G4560) supplemented with 10% foetal bovine serum (Gibco, 10099141 C) and a 1% penicillin-streptomycin solution (Servicebio, G4003); and H460, HCC827 and H1299 cells were cultured in RPMI-1640 medium (Servicebio, G4531) supplemented with 10% foetal bovine serum (Gibco, 10099141 C) and a 1% penicillin-streptomycin solution (Servicebio, G4003). All the cell lines were maintained in a humidified incubator at 37 °C with 5% CO_2_.

### High-throughput transcriptome sequencing

In preliminary studies, a malignantly transformed cell model induced by arsenic treatment (BEAS-2B-As) was successfully established using human bronchial epithelial cells (BEAS-2B) [23]. Subsequently, LC-bio Co., Ltd. (Hangzhou, China) was commissioned to perform high-throughput sequencing of BEAS-2B-As model cells and normal BEAS-2B cells to construct a differential circRNA expression profile. The detailed results are provided in Table S2.

### m5C modification microarray chip

In accordance with the requirements for microarray chip detection, the purity and integrity of the RNA were assessed, and the RNA samples were adjusted for concentration, labelled, and packaged. Subsequently, Aksomics Inc. (Shanghai, China) was commissioned to perform microarray chip detection on adjacent noncancerous tissues and cancerous tissues and successfully constructed a differential circRNA m5C modification profile. The detailed results are provided in Table S3.

### Human tissue samples

The experiment was approved by the Ethics Committee of the First Affiliated Hospital of Guangxi Medical University (No. 2022-KY-E-(289)). Sixty pairs of lung cancer tissues and adjacent noncancer tissues were collected from treatment-naïve patients after written informed consent was obtained. All the samples were nondegraded with good RNA integrity. Based on the relative expression of circRREB1 detected by qPCR, cancer tissue samples with higher circRREB1 expression than the corresponding noncancer tissues were categorized into the high expression group, and those with lower expression were categorized into the low expression group.

### RNA extraction and RT-qPCR

Total RNA was extracted using TRIzol reagent (Invitrogen, 15596018). The RNA concentration was measured with a Nanodrop One spectrophotometer (Thermo Fisher Scientific, USA). Reverse transcription was performed using the GoScript™ Reverse Transcription System (Promega, A5001). qPCR was performed with the GoTaq® qPCR Master Mix (Promega, A6002) kit. The sequences of the primers used in this study are detailed in Table S1.

### Plasmid construction

The overexpression plasmids for NSUN2, HSPA8 and circRREB1, as well as their corresponding empty vectors (pcDNA3.1-3xFlag-C, pCMV-SPORT6 and pcd3.1-circRNA mini, respectively), were constructed by Hunan Fenghui Biotechnology Co., Ltd. (Changsha, China). PINK1 was constructed using pcDNA3.1(+) by the MiaoLing Plasmid Platform (Wuhan, China).

### Construction of a stable silencing system

In this study, we utilized shRNA-containing lentiviral particles purchased from Genechem Co., Ltd. (Shanghai, China) for stable circRREB1 silencing. The viral particles were added to the culture medium of A549 cells. The stably silenced cell lines were selected via monoclonal screening techniques and further cultured for subsequent experiments.

### Cell transfection experiments

Plasmids were transfected in cells at 60–80% confluence using Lipofectamine^TM^ 3000 Reagent (Invitrogen, L3000015) at doses of 2.5 µg/well (6-well) or 0.5 µg/well (96-well), with medium replacement after 8 h. For siRNA transfections, cells at 30% confluence were transfected under serum-free conditions using riboFECT CP Transfection Kit (RiboBio, C10511-05) at volumes of 10 µl/well (6-well) or 1 µl/well (96-well). All transfected cells were cultured for 48 h before experimental analyses.

### Construction of a subcutaneous xenograft tumour model in nude mice

All animal procedures were approved by the Animal Ethics Committee of Guangxi Medical University (No. 202303015). Fifteen female athymic nude mice (4 weeks old) were obtained from the Experimental Animal Center of Guangxi Medical University and housed under specific pathogen-free (SPF) conditions. The mice were randomly divided into 3 groups (*n* = 5), ear-tagged, and weighed. After one week, stably silenced circRREB1 cells or control cells were subcutaneously injected (right flank). The tumour volume (calculated as [length × width^2^]/2) and body weight were recorded every 3 days after xenograft formation. The survival status of the mice was monitored throughout the experiment. Following euthanasia, tumours were excised and processed for the morphological analysis, including fixation with 4% paraformaldehyde for immunohistochemistry.

### RNase R treatment

Total RNA was extracted from cell samples in the absence or presence of 3 U/μg RNase R and incubated at 37 °C for 10 min. The RNase R-treated and untreated RNA samples were subsequently analysed via RT-qPCR.

### Nuclear-cytoplasmic fractionation assay

A total of 1 × 10^7^ cells were collected, and the nuclear and cytoplasmic RNA were isolated using the PARIS™ Kit (Invitrogen, AM1921), followed by washing. RNA was collected after elution.

### Fluorescence in situ hybridization (FISH)

A circRREB1-specific FISH probe (Sangon Biotech, China) and hybridization solution were added, and the mixture was incubated at 42 °C overnight. After hybridization, the slides were washed with a gradient dilution of saline sodium citrate (SSC) and then counterstained with DAPI. An antifade agent was applied to prevent fluorescence quenching, and the cells were imaged using a confocal microscope (Zeiss, Germany).

### Immunofluorescence and fluorescent probe labelling techniques

The cells were fixed with tissue fixative for 15 min, followed by three washes with 1 × PBS. The cells were then blocked with blocking buffer (1 × PBS/5% goat serum/0.3% Triton™ X-100) for 1 h at room temperature. Subsequently, an anti-HSPA8 primary antibody (Proteintech Group, 1:200, 10654-1-AP, RRID: AB_2120153) was added. After the incubation with the primary antibody, the cells were incubated with a fluorescent dye-conjugated secondary antibody. The nuclei were counterstained with DAPI, and antifade reagent was applied to prevent fluorescence quenching. Finally, the cells were imaged with a confocal microscope (Zeiss, Germany).

The subcellular localization of mitochondria and lysosomes was visualized in living A549 cells. The cells were seeded in 12-well chamber slides and incubated for 48 h to allow transfection. The mitochondria and lysosomes were then labelled with the fluorescent probes Mito-Tracker Green (Beyotime, C1048) and Lyso-Tracker Red (Beyotime, C1046) at a concentration of 50 nM for 30 min. The cells were then imaged with a confocal microscope (Zeiss, Germany).

### CCK-8 assay

A Cell Counting Kit-8 (CCK-8) assay (Dojindo, CK04) was utilized to assess cell viability. The CCK-8 reagent was mixed with the culture medium at a ratio of 1:10 and incubated at 37 °C for 90 min. The absorbance of the cells was then measured at a wavelength of 450 nm, and the relative cell viability was calculated.

### EdU assays

A Cell-Light EdU Apollo567 In Vitro Kit (RiboBio, C10310-1) was used to evaluate the cell proliferation capacity. EdU was used to label the cells overnight. The cells were then fixed, and Apollo® and DNA staining were performed. Finally, cell proliferation was analysed with the EVOS® FL Auto Imaging System and ImageJ software.

### Flow cytometry analysis of the cell cycle

A Cell Cycle Detection Kit (KeyGen Biotech, KGA9101) was used to assess the cell cycle. The cells were harvested, rinsed with PBS, and fixed with 70% ethanol. Following fixation, the cells were treated with RNase to digest the RNA and then stained with propidium iodide (PI). The cell cycle distribution was ultimately assessed using a CytoFLEX flow cytometer (Beckman Coulter, USA). The proliferation index (PI) was calculated as (S + G2-M)/(G1 + S + G2-M).

### Flow cytometry analysis of cell apoptosis

An Annexin V-FITC/PI Apoptosis Detection Kit (KeyGen Biotech, KGA1102) was used to assess apoptosis. The cells were digested with trypsin without EDTA and collected. Then, the cells were stained with Annexin V-FITC and propidium iodide. The apoptotic cells were ultimately analysed with a CytoFLEX flow cytometer (Beckman Coulter, USA). Relative cell apoptosis was calculated as the percentage of apoptotic cells in the experimental set/the percentage of apoptotic cells in the control set.

### Cell migration assays

A wound healing assay and a Transwell assay were employed to evaluate the cell migration ability. After creating a scratch using a pipette tip, cells were imaged at 0 h and 24 h with a microscope (Olympus Japan). During this time, cells were cultured in serum-free medium to rule out proliferation effects.

Transwell migration assays were conducted in transwell chambers. Cells in serum-free medium were seeded in the upper chambers, with 700 μl of complete medium used as a chemoattractant in the lower compartments. Following 24 h of incubation (37℃, 5% CO_2_), the migrated cells were fixed and stained with crystal violet. Cells that migrated through the membrane were quantified through EVOS® FL Auto Imaging System coupled with ImageJ analysis.

### RNA stability assay

The cells were treated with 2 µg/ml actinomycin D (Act D) diluted in complete growth medium. Total RNA was extracted at 0 h, 4 h, 8 h, 12 h and 24 h after treatment using TRIzol reagent, and the expression levels of the target genes were assessed via RT-qPCR.

### Protein stabilization assay

The cells were treated with 50 μg/ml cycloheximide (CHX) diluted in complete growth medium. Protein samples were collected at 0 h, 4 h, 8 h and 12 h after treatment. Additionally, cells seeded into 6-well plates were treated with 10 μM MG132 diluted in complete growth medium. Protein samples were collected at 0 h and 24 h. WB was performed to detect the expression levels of the proteins.

### Western blot (WB)

The cells were lysed with a custom cell lysis solution (10 mM Tris-HCl [pH 7.4], 1% SDS, and 1 mM Na_3_VO_4_) to obtain protein, which was heat denatured, ultrasonically fragmented, and centrifuged. The protein concentrations were determined with a Pierce™ BCA Protein Assay Kit (Thermo Fisher Scientific, 23227). The protein samples were then separated on SDS-PAGE gels and transferred to PVDF membranes. The membranes were subsequently incubated with a blocking solution and primary and secondary antibodies and then washed with TBS and TBST. Finally, the target proteins were qualitatively and semiquantitatively analysed using an enhanced chemiluminescence (ECL) detection kit, and images were acquired. The antibodies used in this study were as follows: anti-HSPA8 (Proteintech Group, 1:10000, 10654-1-AP, RRID: AB_2120153), anti-PINK1 (Santa Cruz Biotechnology, 1:500, sc-517353, RRID: AB_2923157), anti-Parkin (Proteintech Group, 1:2000, 14060-1-AP, RRID: AB_2878005), anti-LC3 (Proteintech Group, 1:2500, 14600-1-AP, RRID: AB_2137737), anti-TOM20 (Proteintech Group, 1:10000, 66777-1-Ig, RRID: AB_2919367), anti-β-actin (Proteintech Group, 1:10000, 66009-1-Ig, RRID: AB_2883836), anti-mouse (Proteintech, 1:2500, SA00001-1, RRID: AB_2864335), and anti-rabbit (Proteintech Group, 1:2500, SA00001-2, RRID: AB_2722564).

### RNA immunoprecipitation (RIP)

A RIP kit (IEMed, IEMed-K303) was used for the RIP assay. According to the instructions provided with the kit, circRREB1-protein complexes were enriched using Protein A/G beads. After elution and purification, the RNA was collected and subjected to reverse transcription quantitative polymerase chain reaction (RT-qPCR) to assess the expression levels of circRREB1.

### Methylated RNA immunoprecipitation (MeRIP)

A MeRIP kit (IEMed, IEMed-K368) was used for the MeRIP assay. The protocol for the kit was followed for fractionation, enrichment via Protein A/G beads, elution, and purification of 50 μg of RNA extracted from A549 cells. The RNA obtained after these steps was analysed via reverse transcription quantitative polymerase chain reaction (RT-qPCR) to assess the levels of circRREB1 enrichment.

### Crosslinking-immunoprecipitation (CLIP)

A crosslinking-immunoprecipitation (CLIP) kit (BersinBio, Bes3014) was used to perform the CLIP assay. The cells were subjected to ultraviolet irradiation and then lysed on ice. The lysate was then centrifuged, aliquoted, and labelled. A 3'end adapter was ligated to the RNA, and reverse transcription was performed using the tailing method. The circRREB1 sample was then divided into segments 50 bp in length for the CLIP-qPCR analysis.

### Tagged RNA affinity purification (TRAP)

A TRAP kit (BersinBio, Bes5106) was used to detect proteins that interact with circRREB1. A circRREB1-MS2 fusion expression vector was designed and constructed, and the cells were transfected using the Lipofectamine™ 3000 Transfection Kit. Cells were lysed, and GSH magnetic beads were used to isolate circRREB1-protein complexes. These complexes then underwent further separation and purification. Proteins bound to circRREB1 were subsequently identified via polyacrylamide gel electrophoresis (PAGE), silver staining and liquid chromatography-mass spectrometry (LC-MS) analysis. The detailed results are provided in Table S4.

### Coimmunoprecipitation (Co-IP)

A Co-IP Kit (BersinBio, Bes3011) was used for the Co-IP assay. The cells were lysed thoroughly with cell lysis buffer. Then, the samples were incubated with the target antibody overnight. Protein A/G-MagBeads were added to bind to the antibody. After washing to remove nonspecific binders, the target proteins were eluted and the enrichment efficiency was assessed via WB.

### Detection of the mitochondrial membrane potential via JC-1 staining and TMRE staining

The mitochondrial membrane potential was assessed with a Mitochondrial Membrane Potential Assay Kit (Beyotime, C2006). After the cells were incubated with JC-1 working solution, the cells were subsequently washed with 1 × incubation buffer, and fresh complete medium was added. Cellular fluorescence was assessed via confocal microscopy to visualize changes in the mitochondrial membrane potential.

The mitochondrial membrane potential of the cells was analysed by flow cytometry using TMRE (Beyotime, C2001S). TMRE was diluted 1:1000, and the cells were incubated for 30 min, followed by the flow cytometry analysis.

### Data analysis

Statistical analyses included triplicate biological experiments, and the data are presented as the means ± SDs. Parametric (Student's t test, ANOVA) or nonparametric tests were applied for group comparisons based on the normality of the data, complemented by Pearson’s/Spearman’s correlation analyses, as appropriate. Diagnostic potential was assessed through a receiver operating characteristic (ROC) curve analysis, whereas the Kaplan-Meier methodology was used to evaluate survival outcomes. The analytical workflows utilized SPSS 27.0 (IBM) for hypothesis testing, with visualization implemented in GraphPad Prism 9.0 and R 4.3.2. Significance thresholds were *p < 0.05 and ns = not significant (adjusted for multiple comparisons, where applicable).

## Results

### circRREB1 is significantly upregulated in lung cancer

**Fig. 1:**
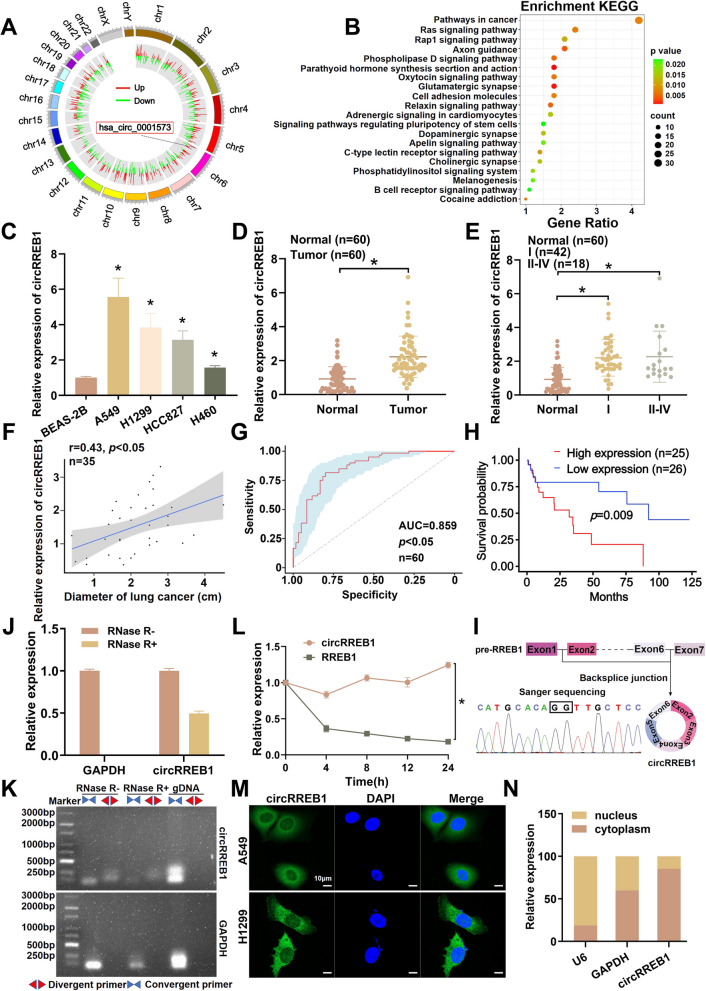
circRREB1 is significantly upregulated in lung cancer. **A** Circular plot of the high-throughput sequencing results for circRNAs. **B** KEGG pathway analysis downstream of circRREB1. **C** qPCR was used to measure circRREB1 expression levels in BEAS-2B, A549, H1299, HCC827 and H460 cells. **D** qPCR was performed to measure circRREB1 expression levels in 60 pairs of lung cancer and adjacent noncancer tissues. **E** qPCR was used to measure circRREB1 expression levels in tumours at different stages. **F** Correlation analysis of circRREB1 expression with the tumour diameter. **G** ROC curve for circRREB1. **H** Survival analysis of circRREB1 expression. **I** Query of the chromosomal information of circRREB1 on the UCSC Genome Browser website, as well as the Sanger sequencing results. **J** After 10 min of RNase R treatment, RT-qPCR was performed to measure the expression of circRREB1 and GAPDH. **K** PCR analysis of the convergernt and divergent primers of circRREB1 in cDNA and gDNA after RNase R treatment (gDNA, genomic DNA). **L** After treatment with Act D (2 μg/ml), qPCR was performed to measure the expression of circRREB1 and the RREB1 mRNA. **M** FISH was performed on A549 and H1299 cells showed that circRREB1 was abundant in the cytoplasm, and DAPI was used to stain the nucleus. Scale bar, 10 μm. **N** Detection of the subcellular localization of circRREB1 via a nuclear-cytoplasmic fractionation experiment

We constructed differential circRNA expression profiles using human bronchial mucosal epithelial cells as the control group and an arsenic-induced malignant cell model as the experimental group to identify circRNAs that play key roles in lung cancer (Fig. 1A) [23]. Through a bioinformatics analysis of highly expressed circRNAs and Kyoto Encyclopedia of Genes and Genomes (KEGG) pathway enrichment analysis, we identified a circRNA closely related to cancer, circRREB1 (Fig. 1B). We subsequently examined circRREB1 expression in BEAS-2B, A549, H1299, HCC827 and H460 cells, and the results of the qPCR experiments suggested that circRREB1 was highly expressed in these lung cancer cell lines (Fig. 1C). Furthermore, circRREB1 expression was assessed in 60 pairs of lung cancer tissues and paracancerous tissues and was significantly upregulated in the lung cancer tissues (Fig. 1D). Concurrently, an in-depth clinical correlation analysis revealed that circRREB1 expression was positively correlated with the tumour grade and tumour diameter (Fig. 1E, F); ROC analysis revealed an area under the curve (AUC) of 0.859 (Fig. 1G). Based on these results, we believe that circRREB1 has the potential to be a diagnostic marker for lung cancer. A subsequent survival analysis revealed that patients with high expression of circRREB1 had a poorer prognosis than those with low expression (Fig. 1H).

We queried the UCSC Genome Browser website to comprehensively characterize circRREB1 and found that circRREB1 is formed by the reverse splicing of exons 2–6 of the RREB1 gene located on chromosome 6, and its circularization site was confirmed by Sanger sequencing (Fig. 1I). Compared with linear RNA, circRNA is resistant to digestion by linear nucleases. After treating the total RNA sample with RNase R, circRREB1 was more resistant to RNase R digestion than linear GAPDH (Fig. 1J). We also designed circRREB1-specific divergent and convergent primers and performed PCR amplification using three groups of samples (RNase R- group, RNase R + group and gDNA group). After agarose gel electrophoresis and imaging, the products amplified by the divergent primers were more resistant to RNase R digestion than those amplified by the convergent primers (Fig. 1K). We subsequently treated the cells with Act D, extracted RNA at 0 h, 4 h, 8 h, 12 h and 24 h, and detected the expression of circRREB1 and RREB1 by qPCR. The results revealed that circRREB1 had a longer half-life than RREB1 (Fig. 1L). Through the aforementioned results, we identified circRREB1 as having a circular structure. Finally, through FISH performed on the lung cell lines A549 and H1299, as well as nuclear-cytoplasmic fractionation assays carried out in A549 cells, we investigated the subcellular localization of circRREB1. The experimental outcomes demonstrated that circRREB1 is predominantly localized to the cytoplasm. (Fig. 1M, N). However, our FISH experiments using BEAS-2B cells revealed that circRREB1 is predominantly localized to the nucleus (Supplementary Fig. S1A).

### m5C modification of circRREB1

**Fig. 2:**
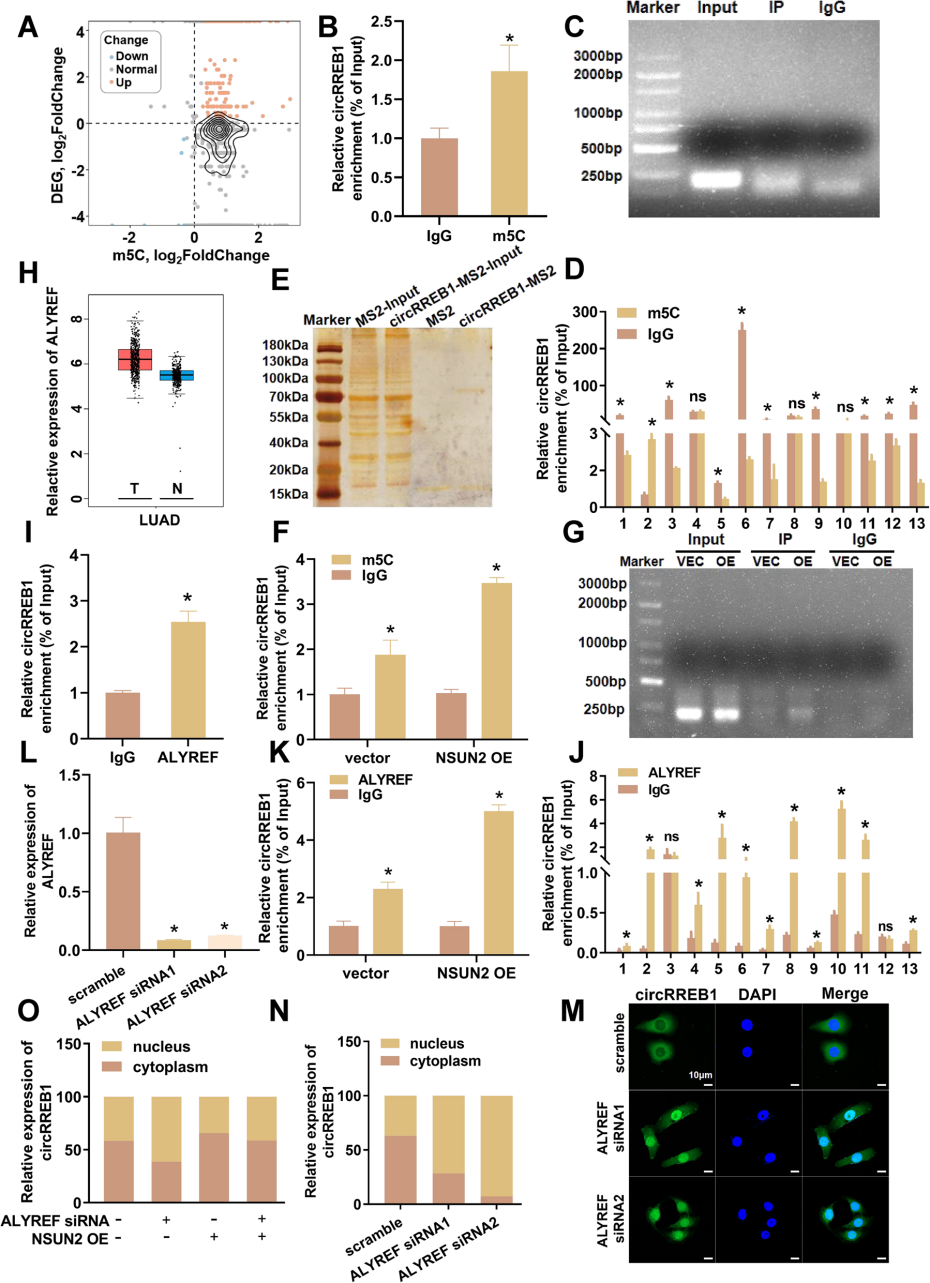
The m5C modification of circRREB1. **A** Scatter plot comparing the variation in each gene according to high-throughput sequencing and m5C microarray chip detection (DEG, differentially expressed genes). **B** MeRIP-qPCR measurement of the m5C modification in circRREB1.** C** Image of the agarose gel electrophoresis MeRIP-qPCR products. **D** CLIP-qPCR was performed to measure m5C-modified regions in circRREB1. **E** Silver staining results for the circRREB1 TRAP experiment. **F** Overexpression of NSUN2 via MeRIP-qPCR to detect m5C modifications in circRREB1. **G** Agarose gel electrophoresis MeRIP-qPCR products (VEC, vector; OE, NSUN2 OE). **H** Expression levels of ALYREF in lung cancer and adjacent noncancerous tissues. (N, nontumorous tissue; T, tumorous tissue). **I** RIP-qPCR was performed to detect the direct binding of circRREB1 with ALYREF. **J** CLIP-qPCR was used to measure the regions of circRREB1 to which ALYREF binds. **K** NSUN2 overexpression and RIP-qPCR was used to detect the interaction between circRREB1 and ALYREF. **L** The silencing efficiency of ALYREF. **M** After ALYREF was silenced, FISH was used to detect the subcellular localization of circRREB1 (green fluorescence: circRREB1; blue fluorescence: nucleus). Scale bar, 10 μm. **N** After ALYREF was silenced, a nuclear-cytoplasmic fractionation experiment was performed to detect the subcellular localization of circRREB1. **O** Overexpression of NSUN2 with silencing of ALYREF and nuclear-cytoplasmic fractionation experiment was performed to detect the subcellular localization of circRREB1

Our preliminary research revealed differences in the subcellular localization of circRREB1 between A549 and BEAS-2B cells. Given that abnormal circRNA expression is usually linked to tumour development, we aimed to explore the reasons for differential circRNA expression in detail. The m5C modification plays a crucial role in regulating the fate of circRNAs. We conducted m5C microarray chip detection using cancerous tissues as the experimental group and adjacent noncancerous tissues as the control group to obtain the differential m5C modification profile. The results suggested that circRREB1 might be modified by m5C (Fig. 2A). We performed MeRIP experiments using m5C modification-specific antibodies and found that the m5C antibody significantly enriched circRREB1, indicating the presence of the m5C modification in circRREB1 (Fig. 2B, C). We subsequently designed specific truncated primers in 50 bp segments and conducted CLIP experiments to explore the sites of the m5C modification on circRREB1. The qPCR results revealed that the m5C modification was located in the second segment (Fig. 2D).

The m5C modification requires the action of methyltransferases (writers). Previous studies indicated that eukaryotic m5C modifications are typically mediated by the NOL1/NOP2/sun (NSUN) family and DNA methyltransferase member 2 (DNMT2) [24]. We initially conducted a TRAP experiment to identify the writer that mediates the m5C modification of circRREB1. After polyacrylamide gel electrophoresis (PAGE) and silver staining, we performed liquid chromatography-mass spectrometry (LC-MS) to identify the proteins bound to circRREB1 (Fig. 2E). Through the LC-MS results, we identified NSUN2, a writer that has been reported to mediate m5C modifications. By querying data from The Cancer Genome Atlas (TCGA) database, we found that NSUN2 is highly expressed in lung cancer tissues and that patients with high NSUN2 expression have a poorer prognosis than those with low NSUN2 expression (Supplementary Fig. S1B, C). This trend is consistent with that identified for the high expression of circRREB1 in lung cancer tissues. Therefore, NSUN2 was selected for further study. We conducted MeRIP experiments after NSUN2 was overexpressed. The results revealed that the m5C modification level of circRREB1 was significantly increased (Fig. 2F, G).

The biological functions of m5C modifications are typically associated with reader proteins. Our TRAP-MS results revealed that the main m5C reader in this study was Aly/REF Export Factor (ALYREF), which has been shown to recognize m5C modifications and regulate RNA stability and nuclear export [25]. Data from TCGA database indicate that ALYREF is highly expressed in lung cancer patients and that high ALYREF expression is associated with a poor prognosis (Fig. 2H, Supplementary Fig. S1D). This trend is consistent with the high expression of circRREB1 in lung cancer tissues and its predominant localization in the cytoplasm. Therefore, ALYREF was selected for further study. We first performed RIP experiments and found that circRREB1 directly binds to ALYREF (Fig. 2I). Subsequent CLIP-qPCR revealed that the binding region of circRREB1 with ALYREF is located in the second segment, which coincides with the m5C modification segment (Fig. 2J). Upon the overexpression of NSUN2, ALYREF was able to enrich more circRREB1 (Fig. 2K). These results suggest that ALYREF can recognize the m5C modification on circRREB1. We designed ALYREF siRNAs to explore the pathways by which ALYREF reportedly regulates the fate of RNA molecules (Fig. 2L). By performing RNA stability experiments, we found that the stability of circRREB1 did not change after ALYREF silencing (Supplementary Fig. S1E). FISH and nuclear-cytoplasmic fractionation experiments revealed that the nuclear content of circRREB1 increased significantly after ALYREF silencing (Fig. 2M, N). The finding that silencing ALYREF increases the nuclear expression of circRREB1 aligns with the elevated expression of ALYREF in lung cancer tissues. We also found that NSUN2 overexpression significantly increased the nuclear export of circRREB1, and ALYREF silencing in these cells reversed the increase in nuclear export induced by NSUN2 overexpression (Fig. 2O). Collectively, these results indicate that NSUN2 increases the m5C modification level of circRREB1 and that ALYREF can recognize the m5C modification of circRREB1 and regulate the nuclear export of circRREB1 in an m5C-dependent manner.

### circRREB1 significantly promotes lung cancer progression in vitro

**Fig. 3:**
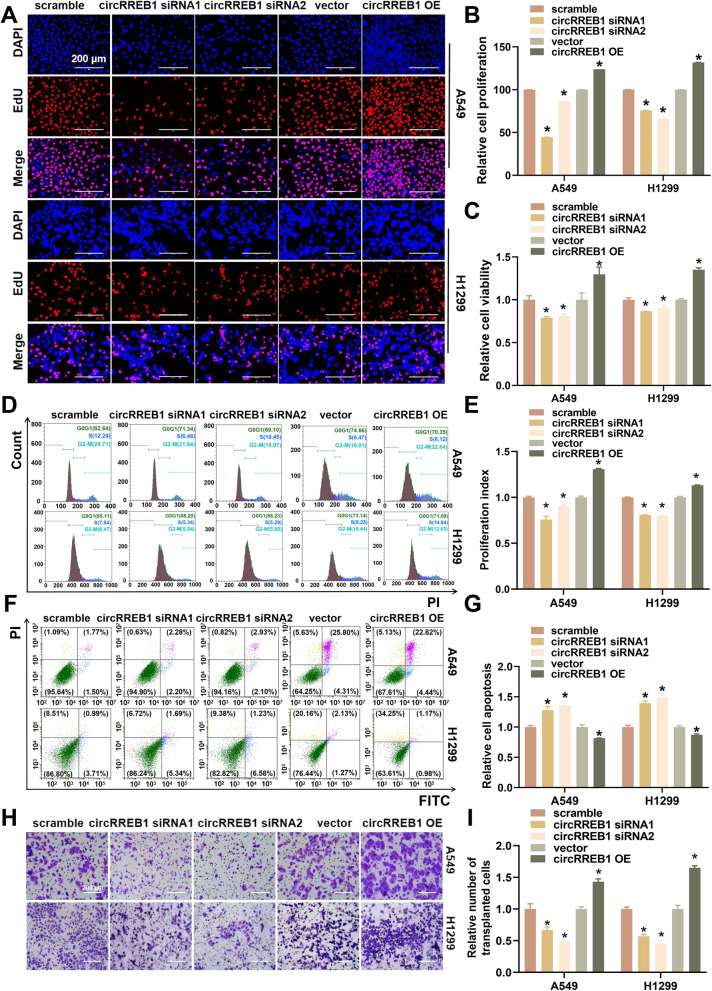
circRREB1 significantly promotes lung cancer development in vitro. **A** An EdU assay was performed to detect cell proliferation after the transient silencing and overexpression of circRREB1. Scale bar, 200 μm. **B** Analysis of the EdU assay results. **C** A CCK-8 assay was performed to assess cell viability following the transient silencing and overexpression of circRREB1. **D** Flow cytometry was used to analyse the cell cycle upon the transient silencing and overexpression of circRREB1. **E** Analysis of the cell cycle data obtained via flow cytometry. **F** Flow cytometry was used to evaluate apoptosis after the transient silencing and overexpression of circRREB1. **G** Analysis of apoptosis data obtained via flow cytometry. **H** Transwell assays were used to assess cell migration after the transient silencing and overexpression of circRREB1. Scale bar, 200 μm. **I** Analysis of the Transwell migration assay data

We constructed circRREB1 silencing and overexpression systems to explore the biological functions of circRREB1 **(**Supplementary Fig. S2A). EdU assays revealed that cell proliferation decreased upon circRREB1 silencing but increased upon circRREB1 overexpression (Fig. 3A, B). CCK-8 assays revealed that cell viability decreased after circRREB1 silencing but increased after circRREB1 overexpression (Fig. 3C). A flow cytometry analysis of the cell cycle revealed that cell cycle progression was inhibited after circRREB1 silencing but accelerated after circRREB1 overexpression (Fig. 3D, E). Flow cytometry assays revealed that apoptosis increased after circRREB1 silencing but decreased after circRREB1 overexpression (Fig. 3F, G). Transwell and wound healing assays revealed that cell migration decreased after circRREB1 silencing but increased after circRREB1 overexpression (Fig. 3H, I; Supplementary Fig. S2B, C). In summary, circRREB1 can significantly promote the progression of lung cancer in vitro. ​To further elucidate the impact of m5C modification on the biological functions of circRREB1, we simultaneously silenced the reader protein ALYREF and overexpressed circRREB1, then assessed cell viability and migration ability. circRREB1 overexpression rescued the reduction in cell viability and migration ability induced by ALYREF silencing (Supplementary Fig. S2D–F).​

### circRREB1 significantly promotes lung cancer progression in vivo

**Fig. 4:**
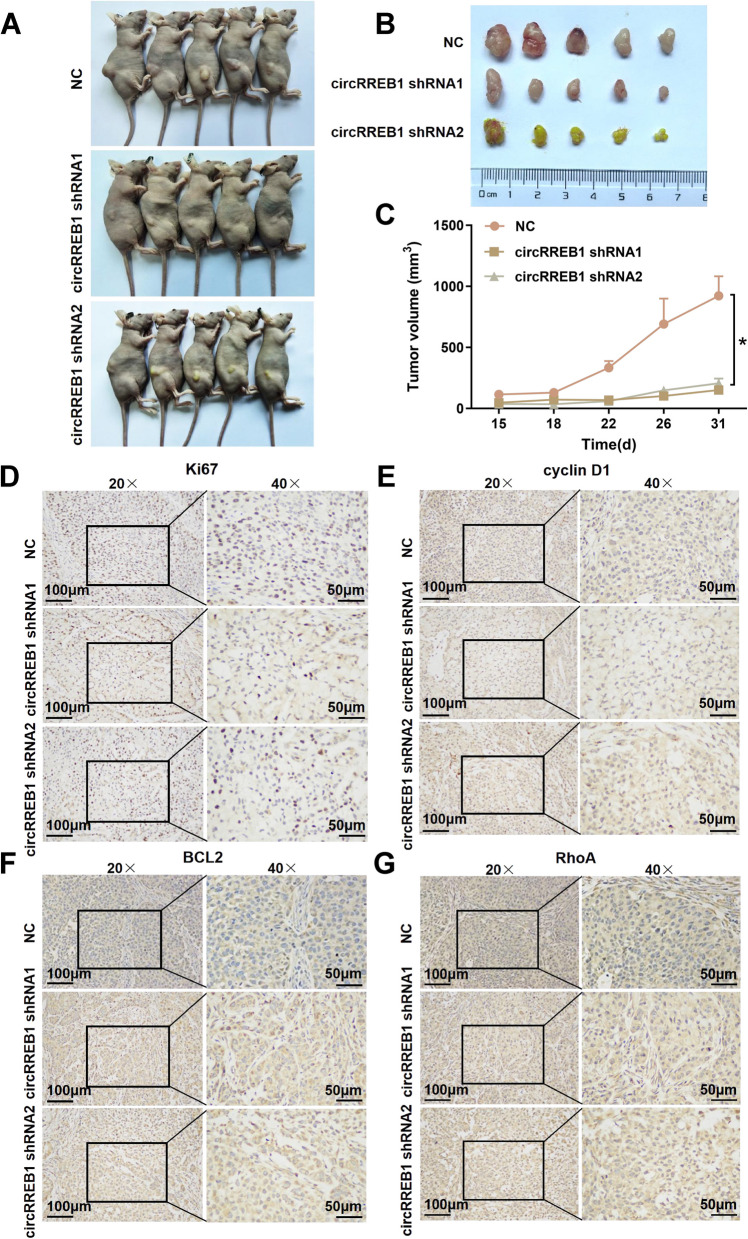
circRREB1 significantly promotes lung cancer progression in vivo.** A** Cell lines with stable circRREB1 silencing were injected into the right flank of four-week-old nude mice, and tumour growth was observed over four weeks. (Five nude mice were analysed per group). **B** After the nude mice were euthanized, the tumours were excised and photographed. **C** Analysis of tumour growth curves in nude mice. **D** Immunohistochemical analysis of Ki67 expression in the tumour tissues of nude mice (left panel: 20 × magnification, scale bar 100 μm; right panel: 40 × magnification, scale bar 50 μm). **E** Immunohistochemical analysis of cyclin D1 expression in the tumour tissues of nude mice (left panel: 20 × magnification, scale bar 100 μm; right panel: 50 × magnification, scale bar 40 μm). **F** Immunohistochemical analysis of BCL2 expression in the tumour tissues of nude mice (left panel: 20 × magnification, scale bar 100 μm; right panel: 40 × magnification, scale bar 50 μm). **G** Immunohistochemical analysis of RhoA expression in the tumour tissues of nude mice (left panel: 20 × magnification, scale bar 100 μm; right panel: 40 × magnification, scale bar 50 μm)

We elucidated the in vitro functions of circRREB1 in lung cancer, and thus we next explored its in vivo functions. First, we successfully constructed a stable circRREB1-silenced A549 cell line (Supplementary Fig. S2G, H). Control cells and stable circRREB1-silenced cells were subcutaneously injected into the right flanks of nude mice. After tumour formation, the survival status of the nude mice was observed every three days, and the body weight and tumour volume were assessed and recorded for each nude mouse. At the end of the observation period, the nude mice were euthanized, and the tumour tissues were harvested and photographed (Fig. 4A, B). A statistical analysis of tumour growth was performed, and the results revealed that tumour growth was slower after circRREB1 silencing (Fig. 4C). We conducted immunohistochemical experiments to further clarify the biological functions of circRREB1 in vivo and found that in tumour tissues with stable circRREB1 silencing, the expression levels of the proliferation-related protein Ki67, the cycle-related protein cyclin D1, the apoptosis-related protein BCL2, and the migration-related protein RhoA were reduced (Fig. 4D‒G, Supplementary Fig. S2I‒L). In summary, these results indicate that circRREB1 can significantly promote the progression of lung cancer in vivo.

### circRREB1 regulates mitochondrial function through mitophagy

**Fig. 5:**
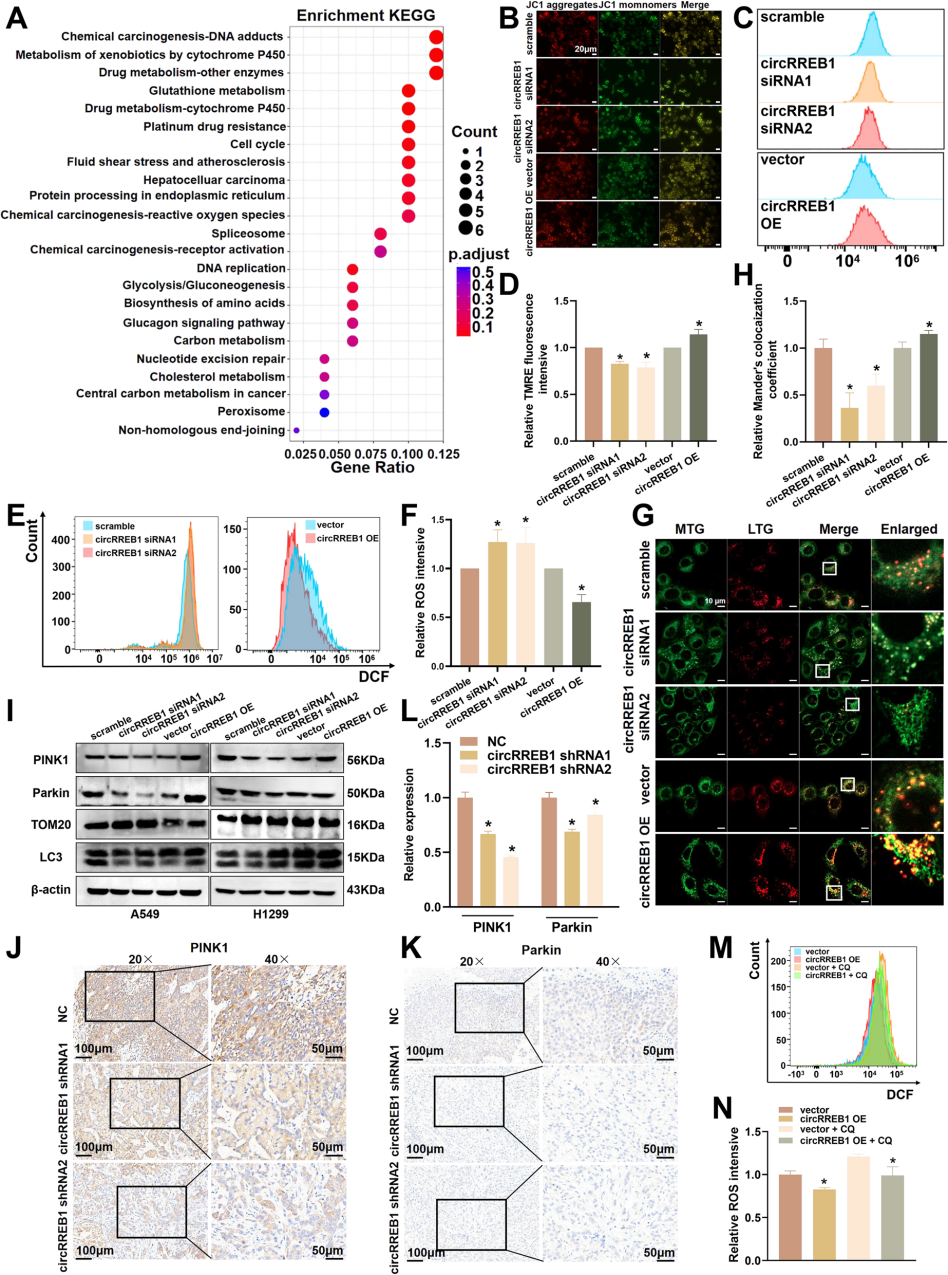
circRREB1 regulates mitochondrial function through mitophagy.** A** KEGG enrichment analysis of the circRREB1 TRAP-MS results. **B** JC-1 detection of the mitochondrial membrane potential after the transient silencing and overexpression of circRREB1. Scale bar, 20 μm. **C** Flow cytometry detection of TMRE fluorescence intensive after the transient silencing and overexpression of circRREB1. **D** Statistical analysis of TMRE fluorescence intensive after the transient silencing and overexpression of circRREB1. **E** Flow cytometry detection of intracellular ROS levels after the transient silencing and overexpression of circRREB1. **F** Statistical analysis of the intracellular ROS levels after the transient silencing and overexpression of circRREB1. **G** Mitochondrial-lysosomal colocalization after the silencing and overexpression of circRREB1. Scale bar, 10 μm. MTG: MitoTracker Green; LTR: LysoTracker Red. **H** Relative Mander's colocalization coefficients of mitochondrial/lysosomal after the silencing and overexpression of circRREB1. **I** WB was performed to detect the expression levels of PINK1, Parkin, LC3 and TOM20 after the transient silencing and overexpression of circRREB1. **J** Immunohistochemical analysis of PINK1 expression in the tumour tissues of nude mice (left panel: 20 × magnification, scale bar 100 μm; right panel: 40 × magnification, scale bar 50 μm). **K** Immunohistochemical analysis of Parkin expression in the tumour tissues of nude mice (left panel: 20 × magnification, scale bar 100 μm; right panel: 40 × magnification, scale bar 50 μm). **L** ​​Quantitative immunohistochemistry analysis of PINK1 and Parkin expression.​ **M** Detection of intracellular ROS levels after the addition of chloroquine (50 μM) to cells overexpressing circRREB1. **N** Statistical analysis of the intracellular ROS levels after the addition of chloroquine (50 μm) to cells overexpressing circRREB1

Through in vitro and in vivo functional experiments, we found that circRREB1 can promote the development of lung cancer. However, the molecular mechanisms by which circRREB1 promotes the development of lung cancer still need further exploration. We performed a KEGG pathway enrichment analysis on the TRAP-MS results and identified several pathways related to the cytochrome P450 enzyme system (Fig. 5A). Studies have shown that metabolites produced via the metabolism of various drugs or chemical toxins by cytochrome P450 enzymes can cause mitochondrial dysfunction [26, 27]. Therefore, we assessed mitochondrial function. Through JC-1 staining, we detected a decrease in the level of JC-1 aggregates (indicated by red fluorescence) and an increase in the level of JC-1 monomers (indicated by green fluorescence) after circRREB1 was silenced, indicating a decrease in the mitochondrial membrane potential. In contrast, overexpression of circRREB1 led to an increase in the level of JC-1 aggregates and a decrease in the level of JC-1 monomers, indicating an increase in the mitochondrial membrane potential (Fig. 5B). Additionally, flow cytometry was performed after TMRE staining to detect the mitochondrial membrane potential. These results were consistent with those of JC-1 staining, which revealed that silencing circRREB1 reduced the mitochondrial membrane potential, whereas overexpressing circRREB1 increased the mitochondrial membrane potential (Fig. 5C, D). We also measured the intracellular reactive oxygen species (ROS) levels and found that the ROS levels increased after circRREB1 was silenced but decreased after circRREB1 was overexpressed, suggesting that mitochondrial function was impaired after circRREB1 was silenced and increased after circRREB1 was overexpressed (Fig. 5E, F).

Mitophagy, the process by which autophagy specifically targets damaged mitochondria, is a key pathway for regulating mitochondrial function. We used MitoTracker Green and LysoTracker Red to label mitochondria and lysosomes, respectively, and observed the colocalization of mitochondria with lysosomes to explore whether circRREB1 regulates mitophagy. We found that silencing circRREB1 significantly reduced the colocalization of mitochondria with lysosomes, while overexpression of circRREB1 significantly increased this colocalization, suggesting that circRREB1 can positively regulate mitophagy (Fig. 5G, H). The PINK1/Parkin pathway is a classical pathway for mitophagy. We conducted WB to detect proteins related to PINK1-mediated mitophagy. The results showed that silencing circRREB1 reduced the expression of PINK1, Parkin, and LC3 and increased the expression of the mitochondrial outer membrane protein TOM20. In contrast, overexpression of circRREB1 increased the expression of PINK1, Parkin, and LC3 and decreased TOM20 expression, indicating that circRREB1 can regulate mitophagy at the cellular level (Fig. 5I, Supplementary Fig. S3A–D). We explored whether circRREB1 promotes mitophagy in vivo by performing IHC on xenografts to assess PINK1 and Parkin levels and found that silencing circRREB1 reduced their expression (Fig. 5J‒L). We subsequently extracted proteins from the tumour tissues of nude mice and conducted WB to detect mitophagy-related proteins. Consistent with the IHC results, stable silencing of circRREB1 significantly reduced the expression of mitophagy-related proteins (Supplementary Fig. S3E). When we simultaneously silenced the m5C reader ALYREF and overexpressed circRREB1, mitochondrial-lysosomal co-localization showed that m5C modification also regulates the resulting increase in co-localization caused by circRREB1 overexpression (Supplementary Fig. S3F, G).​ When we overexpressed circRREB1, treated cells with the autophagy inhibitor chloroquine (CQ), and detected ROS levels, we found that the addition of chloroquine inhibited autophagy, leading to an increase in ROS levels, indicating an increase in the number of damaged mitochondria (Fig. 5M, N). These results suggest that circRREB1 enhances mitochondrial function through mitophagy. We added chloroquine to inhibit mitophagy and detected the level of apoptosis to explore whether circRREB1 regulates the development of lung cancer through mitophagy and found that the addition of chloroquine reversed the decrease in apoptosis induced by the overexpression of circRREB1 (Supplementary Fig. S3H, I). In summary, circRREB1 may regulate mitochondrial function through PINK1/Parkin-mediated mitophagy and thereby promote the development of lung cancer.

### circRREB1 binds to the HSPA8 protein, inhibiting its ubiquitination and degradation

**Fig. 6:**
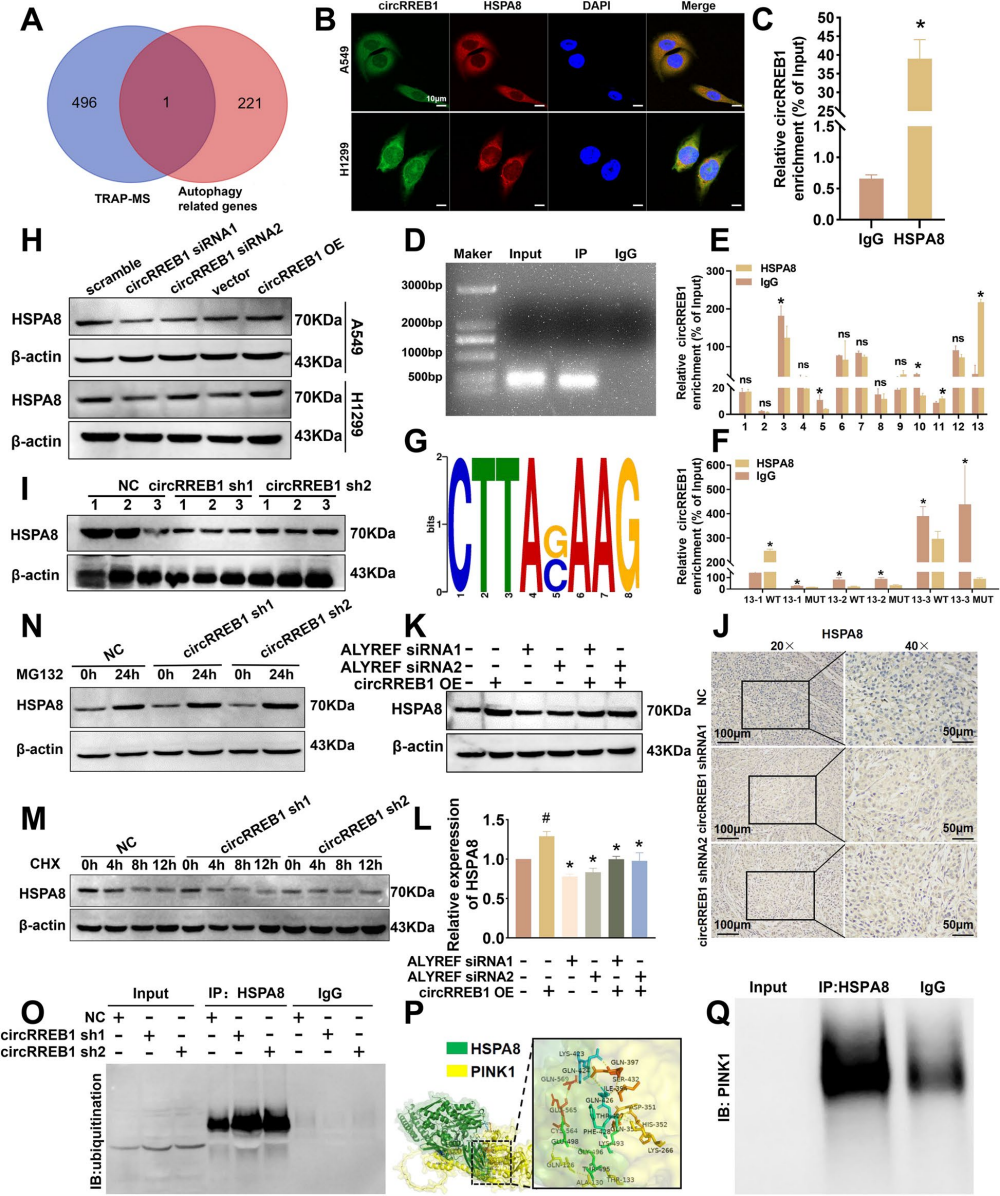
circRREB1 binds to the HSPA8 protein, inhibiting its ubiquitination and degradation.** A** Venn diagram of the TRAP-MS data and autophagy-related genes. **B** Detection of circRREB1 and HSPA8 colocalization via FISH and IF. Scale bar, 10 μm. **C** RIP-qPCR was performed to detect the direct binding of circRREB1 to HSPA8. **D** Agarose gel electrophoresis of RIP-qPCR products​. **E** CLIP-qPCR was used to measure the regions of circRREB1 to which HSPA8 binds. **F** CLIP-qPCR was used to measure the motif of circRREB1 to which HSPA8 binds. **G ​​**circRREB1 binding motif on HSPA8​. **H** WB was used to detect the HSPA8 protein expression level after the silencing and overexpression of circRREB1. **I** WB was performed to detect the HSPA8 protein expression level in tumour tissues from nude mice. **J** Immunohistochemical analysis of HSPA8 expression in tissues from nude mice (left panel: 20 × magnification, scale bar 100 μm; right panel: 40 × magnification, scale bar 50 μm). **K** After ALYREF silencing and circRREB1 overexpression, WB was used to detect the HSPA8 protein expression level. **L** Quantitative analysis of the WB analysis of HSPA8 protein expression after ALYREF silencing and circRREB1 overexpression. **M** After treatment with CHX (50 μg/ml), WB was performed to detect the HSPA8 protein expression level. **N** After treatment with MG132 (10 μM), WB was performed to detect the protein expression level of HSPA8 in cells. **O** Co-IP was performed to detect the binding of the HSPA8 protein to ubiquitinated proteins. **P** Molecular docking simulation of the interaction of HSPA8 with PINK1. **Q** Co-IP was used to detect the interaction between HSPA8 and PINK1

circRREB1 can regulate PINK1/Parkin-mediated mitophagy, yet the TRAP-MS results for circRREB1 did not include the PINK1 protein, and silencing and overexpressing circRREB1 did not significantly alter PINK1 mRNA levels (Supplementary Fig. S4A). We hypothesize that circRREB1 might regulate PINK1 expression at the posttranscriptional level through intermediary proteins. Therefore, we intersected autophagy-related genes with proteins identified via TRAP-MS to create a Venn diagram and selected the HSPA8 protein (Fig. 6A). The level of the HSPA8 mRNA did not change after the silencing and overexpression of circRREB1 (Supplementary Fig. S4B). Thus, we speculate that circRREB1 might directly interact with the HSPA8 protein to affect its expression. We performed FISH and IF experiments and found that circRREB1 and HSPA8 colocalized (Fig. 6B). Compared with IgG, HSPA8 bound more circRREB1 in the RIP experiments (Fig. 6C, D). These results suggest that circRREB1 directly binds to HSPA8. To define the binding regions between circRREB1 and HSPA8, we performed CLIP assays. Through CLIP experiments, we discovered that HSPA8 binds to region thirteenth of circRREB1 (Fig. 6E). ​​Subsequently, using the ​​RBPMap​​ (http://rbpmap.technion.ac.il/), we predicted potential protein-binding sites in thirteenth segment of circRREB1 and designed ​​segment-specific primers​​ along with ​​corresponding mutagenic primers​​ targeting these binding regions. CLIP experimental results confirmed a specific binding motif between circRREB1 and HSPA8 (Fig. 6F, G).

We subsequently conducted WB experiments to detect the expression of the HSPA8 protein and found that circRREB1 could positively regulate the expression of the HSPA8 protein (Fig. 6H, Supplementary Fig. S4C). The WB analysis of proteins extracted from tumour tissues from nude mice revealed a decrease in HSPA8 expression in the group with stable silencing of circRREB1 (Fig. 6I). Through immunohistochemistry, we found that its expression level was significantly reduced after the stable silencing of circRREB1 (Fig. 6J, Supplementary Fig. S4D). By querying TCGA database, we found that HSPA8 expression is increased in lung cancer patients and that the group with high expression of HSPA8 has a poorer survival rate than the group with low expression, which is consistent with the role of circRREB1 as an oncogenic factor (Supplementary Fig. S4E, F). Since ALYREF can act as a reader protein for the m5C modification of circRREB1 to regulate the nuclear export of circRREB1, we overexpressed circRREB1 while silencing ALYREF. After ALYREF was silenced, the nuclear export of circRREB1 decreased and HSPA8 expression was reduced, as determined by WB; notably, these changes were reversed by circRREB1 overexpression (Fig. 6K, L).

Many studies have shown that circRNAs can regulate protein expression through the ubiquitin-proteasome pathway [28, 29]. First, we treated cells with CHX to inhibit protein synthesis. Compared with the control group, the circRREB1-silenced group presented poorer stability and a shorter half-life of the HSPA8 protein (Fig. 6M, Supplementary Fig. S4G). When we added the proteasome inhibitor MG132, we found that circRREB1 could not regulate the expression of HSPA8 (Fig. 6N). We performed a Co-IP experiment to further investigate the effect of circRREB1 on the level of ubiquitinated HSPA8 and found that the level of ubiquitinated HSPA8 was significantly increased in circRREB1-silenced cells (Fig. 6O). Through the aforementioned experiments, we elucidated that circRREB1 can target HSPA8 and regulate its expression by controlling its ubiquitination. We conducted molecular docking prediction and Co-IP experiments to determine whether HSPA8 directly binds to PINK1, and the results revealed that HSPA8 can directly bind to PINK1 (Fig. 6P, Q).

### circRREB1 regulates PINK1/Parkin-mediated mitophagy via HSPA8, promoting the progression of lung cancer

**Fig. 7:**
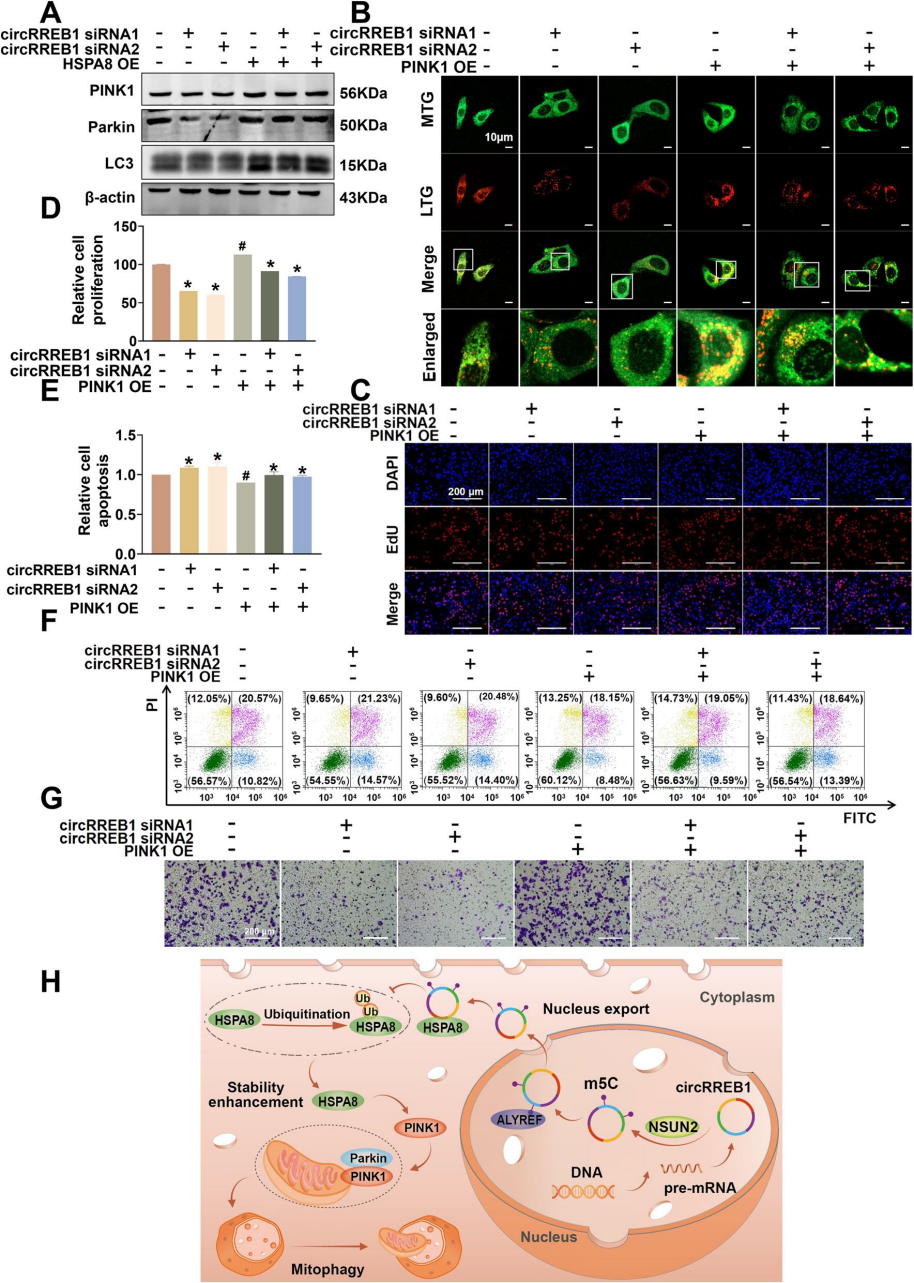
circRREB1 regulates PINK1/Parkin-mediated mitophagy via HSPA8, promoting the progression of lung cancer.** A** WB was used to detect the expression of PINK1, Parkin, and LC3 when circRREB1 was silenced and HSPA8 was overexpressed. **B** Mitochondrial-lysosomal colocalization was detected when circRREB1 was silenced and PINK1 was overexpressed. Scale bar, 10 μm. MTG: MitoTracker Green; LTR: LysoTracker Red. **C** An EdU assay was performed to detect cell proliferation when circRREB1 was silenced and PINK1 was overexpressed. Scale bar, 200 μm. **D** Statistical analysis of the EdU assay results. **E** Statistical analysis of the results of the apoptosis assay. **F** Flow cytometry was performed to detect apoptosis when circRREB1 was silenced and PINK1 was overexpressed. **G** Transwell assays were used to detect migration when circRREB1 was silenced and PINK1 was overexpressed. **H** Graphical abstract

Our previous research revealed that circRREB1 can target and regulate the expression of the HSPA8 protein and that HSPA8 can directly bind to PINK1. We designed rescue experiments to explore whether circRREB1 regulates PINK1 protein expression through HSPA8, which in turn regulates mitophagy to promote lung cancer development. We silenced circRREB1 while overexpressing HSPA8 to detect proteins related to PINK1/Parkin-mediated mitophagy. The results of the WB experiments revealed that HSPA8 overexpression reversed the decrease in the expression of the mitophagy-related proteins PINK1, Parkin, and LC3 induced by the silencing of circRREB1 (Fig. 7A, Supplementary Fig. S4H–J).

We then silenced circRREB1 while overexpressing PINK1 and performed a series of functional assays to explore the ability of circRREB1 to promote lung cancer development through PINK1. First, we detected the level of mitophagy in cells by assessing the colocalization of mitochondria with lysosomes. The results showed that PINK1 overexpression reversed the decrease in mitophagy induced by the silencing of circRREB1 (Fig. 7B, Supplementary Fig. S4K). EdU assays were performed to detect the cell proliferation capacity, and we found that overexpressing PINK1 reversed the decrease in the cell proliferation capacity induced by the silencing of circRREB1 (Fig. 7C, D). Flow cytometry was used to detect cell apoptosis, and the results showed that PINK1 overexpression reversed the increase in cell apoptosis induced by the silencing of circRREB1 (Fig. 7E, F). Transwell assays were performed to detect the cell migration capacity, and the results showed that PINK1 overexpression reversed the decrease in the cell migration capacity induced by the silencing of circRREB1 (Fig. 7G, Supplementary Fig. S4L).

In summary, circRREB1 may increase the expression level of PINK1 by targeting HSPA8, thereby inducing mitophagy and ultimately promoting the development of lung cancer (Fig. 7H).

## Discussion

Lung cancer is the most common malignant tumour and a leading cause of cancer-related death; thus, it poses a serious threat to human life and health. In recent years, the rapid development of targeted therapy has brought new hope to patients, but challenges, such as a lack of targetable genes and gene mutations, still persist [30, 31]. Therefore, the identification of more conserved and specific new targets is particularly important. Our study demonstrated that m5C-modified circRREB1 plays a crucial role in lung cancer progression, providing novel molecular insights into lung cancer development and identifying potential therapeutic targets for its treatment.

As most circRNAs reside in the cytoplasm to perform their key biological functions, elucidating the molecular mechanisms of circRNA nuclear export is critical. Recent studies have shown that circRNAs can be exported via the classic nuclear export pathway mediated by Ran-GTP, exportin-2, and IGF2BP1 [32]. For the first time, this study revealed that circRREB1 undergoes nuclear export in an m5C modification-dependent manner via ALYREF. These findings not only add to the understanding of circRNA nuclear export mechanisms but also offer crucial insights into the diversity of circRNA nucleocytoplasmic transport. m5C modifications can be found on tRNAs, rRNAs, mRNAs, and eRNAs and play crucial roles in regulating RNA stability, translation efficiency, and nucleocytoplasmic transport [14, 25, 33, 34]. However, the molecular mechanisms of the m5C modification in circRNA regulation remain unclear. Our study revealed that the m5C modification of circRREB1 is mediated by the methyltransferase NSUN2 and identified ALYREF as a circRNA m5C reader for the first time. ALYREF is upregulated in lung cancers [35]. Correspondingly, our experimental results demonstrated that circRREB1 is predominantly located in the nucleus of normal human bronchial epithelial cells, whereas it is primarily found in the cytoplasm of lung cancer cell lines.​ These findings provide compelling evidence that the dysregulation of the m5C modification contributes to the altered molecular fate of circRREB1 in lung cancer. Rescue experiments in which ALYREF silencing reversed the effects of circRREB1 overexpression further confirmed the causal link between this pathway and the tumour-promoting function of circRREB1. ​​While ALYREF is an established m5C reader critical for mRNA stability and nuclear export [25, 36], notably, in contrast to its well-established role in regulating mRNA stability, ALYREF promotes circRREB1 nuclear export without affecting its stability, revealing a distinct functional role for ALYREF in circRNA biology. As a core component of the TREX complex with a classical RNA-binding domain, we hypothesize that ALYREF specifically recognizes m5C-modified circRREB1, recruits the TREX complex [37], and facilitates its export via this canonical pathway, a model that requires further validation. In summary, our study shows for the first time the crucial role of the m5C modification in regulating circRNA molecular fate, offering a new theoretical perspective on circRNA expression regulation.

circRNAs primarily serve regulatory functions in life activities and can participate in various cellular events, such as programmed cell death, lipid metabolism and autophagy [38–40]. Mitophagy is an essential regulatory mechanism for maintaining mitochondrial quality and homeostasis. Under normal physiological conditions, mitophagy can identify and eliminate dysfunctional mitochondria in cells in a timely manner, provide sufficient raw materials for the generation of new mitochondria, ensure the energy supply of cells, and thus maintain cellular homeostasis [41]. Mitophagy can be divided into two pathways: ubiquitin-mediated mitophagy and receptor-mediated mitophagy. Ubiquitin-mediated mitophagy includes the PINK1/Parkin pathway and other ubiquitin-mediated pathways, whereas receptor-mediated mitophagy pathways include BCL-2 and adenovirus E1B 19 kDa protein 3 (BNIP3)-mediated mitophagy, FUNDC1-mediated mitophagy, and lipid-mediated mitophagy [42–44]. Compared with receptor-mediated mitophagy pathways, ubiquitin-mediated mitophagy plays a more extensive and critical regulatory role within the cell. Ubiquitin is a ubiquitous protein modifier that marks mitochondria for degradation by binding to target proteins, thereby guiding the formation and maturation of autophagosomes. This process involves multiple important signalling pathways and regulatory factors, such as PINK1 and Parkin, which play key roles in the pathogenesis of various diseases. In contrast, receptor-mediated mitophagy typically relies on specific receptor proteins, and its regulatory mechanism is relatively singular, with limited breadth and depth of research. Therefore, in this study, the ubiquitin-mediated mitophagy pathway was the main focus. In the process of ubiquitin-mediated mitophagy, in addition to the classic PINK1/Parkin pathway, proteins such as MUL1 and ARIH1 also play important regulatory roles [45, 46]. While the PINK1/Parkin pathway depends on the activation of PINK1, the function of MUL1 does not; conversely, MUL1 directly mediates the ubiquitination of mitochondrial proteins, promoting the initiation of mitophagy. ARIH1, on the other hand, can rapidly respond to cellular stress conditions by mediating the ubiquitination of mitochondrial proteins. Since the PINK1/Parkin pathway is the most classical signalling pathway for mitophagy, we first assayed PINK1/Parkin after discovering that circRREB1 can induced mitophagy. The results showed that circRREB1 can activate the PINK1/Parkin signalling pathway, promoting the initiation of mitophagy.

Recent research on the regulation of mitophagy by circRNAs is still limited, and our research indicates that circRREB1 can regulate mitophagy via HSPA8. HSPA8, a molecular chaperone protein, plays essential roles in heat shock, oxidative stress, protein misfolding, and aggregation. Studies have shown that circRNAs can regulate the levels of the posttranslational modifications of RNA-binding proteins (RBPs), including common protein modifications such as phosphorylation, acetylation, ubiquitination, and lipidation. These modifications can significantly affect the function, activity, stability, and localization of proteins. In this study, after treatment with CHX and the proteasome inhibitor MG132, we performed WB to determine whether circRREB1 inhibits the degradation of HSPA8. We focused on ubiquitination, one of the most common mechanisms of protein degradation. Co-IP experiments revealed that circRREB1 inhibits the ubiquitination of HSPA8, thereby inhibiting its degradation. In subsequent experiments, we revealed that HSPA8 binds to PINK1, which then recruits the Parkin protein to induce mitophagy. Our study explored the molecular mechanisms of HSPA8-mediated mitochondrial homeostasis during stress responses, such as the response to oxidative stress, providing new insights. Given the key role of mitophagy in pathological processes such as neurodegeneration, ageing, and cardiovascular diseases, the circRREB1-HSPA8 axis is a vital regulator of mitochondrial health. Its dysfunction may be closely linked to disease development and progression, showing significant research potential and promise as a therapeutic target [47, 48].

In summary, we identified m5C-modified circRREB1, which is significantly overexpressed in lung cancer tissues and cell lines and is associated with a poor prognosis. NSUN2 mediates circRREB1 m5C methylation. Here, we first identified ALYREF as a circRNA m5C reader that recognizes m5C-modified circRNAs and promotes their nuclear export in an m5C-dependent manner. The results from in vitro and in vivo experiments indicate that circRREB1 can significantly promote the progression of lung cancer. Mechanistically, m5C-modified circRREB1 stabilizes the HSPA8 protein by inhibiting its ubiquitination, thereby increasing the expression of PINK1, initiating mitophagy, and ultimately promoting the progression of lung cancer. These new findings not only expand our understanding of the regulatory mechanisms of circRNAs but also provide new directions for the exploration of diagnostic and therapeutic strategies for lung cancer.

## Supplementary Information

Supplementary Material 1.

Supplementary Material 2.

## Data Availability

The datasets used or analyzed in the present study are available from the corresponding author on reasonable request. The circRNA sequencing results have been uploaded to the GEO database (PRJNA971588). The results of the m5C-modified microarray have been uploaded to the GEO database (GSE232282).
