# Supplementary material for: m5C-modified circRREB1 promotes lung cancer progression by inducing mitophagy
Source: J Exp Clin Cancer Res. 2025 Jul 14;44:203. doi: 10.1186/s13046-025-03460-1 (PMC12257754; doi:10.1186/s13046-025-03460-1)
Supplement: Supplementary file 1 — Supplementary Material 1. [file 13046_2025_3460_MOESM1_ESM.pdf]

Supplementary Fig. S1

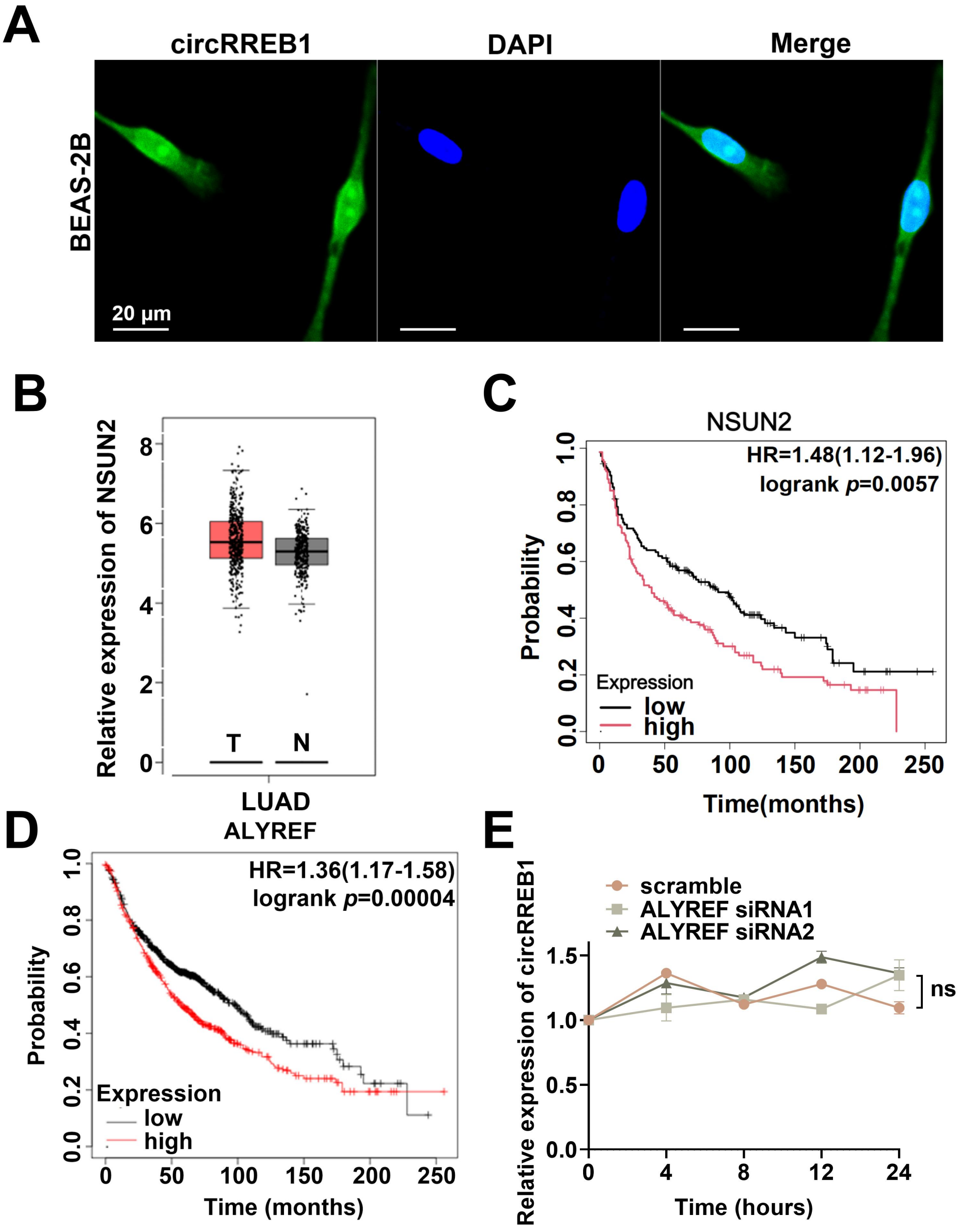

Supplementary Fig. S1 circRREB1 m5C modification relative research. (**A)** FISH was performed on BEAS-2B cells showed that circRREB1 was abundant in the nucleus, and DAPI was used to stain the nucleus. Scale bar, 20 μM. **(B)** Expression of NSUN2 in lung cancer and adjacent non-cancerous tissues. (N, nontumorous tissue; T, tumorous tissue). **(C)** Survival curve analysis for NSUN2 expression. (**D)** Survival curve analysis for ALYREF expression. (**E)** After treatment with Act D (2 μg/ml), qPCR was performed to measure the expression of circRREB1 .

Supplementary Fig. S2

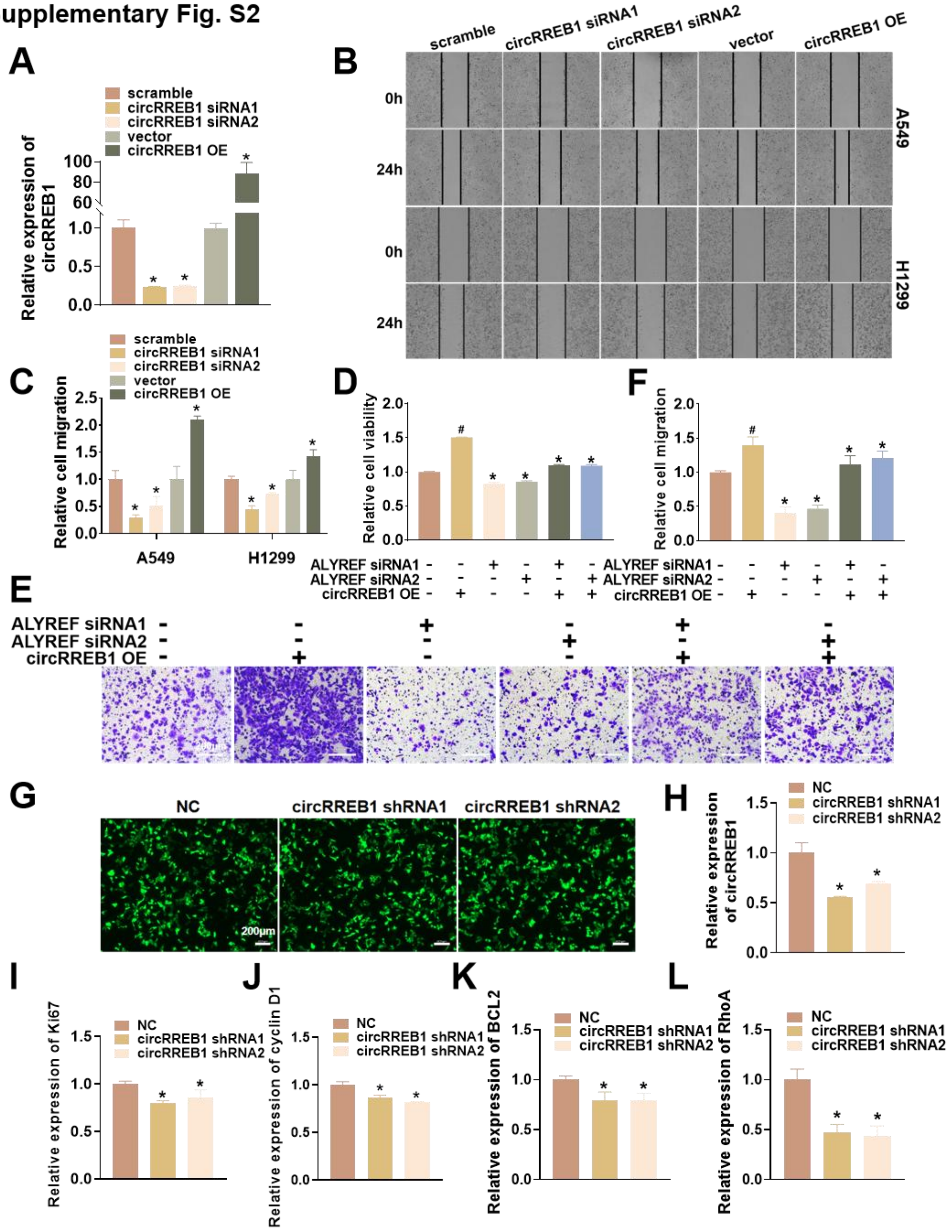

Supplementary Fig. S2 Silencing and overexpression of circRREB1 for in vitro and in vivo functional assays. (A) qPCR was used to measure circRREB1 expression levels following transient silencing and overexpression of circRREB1. **(B)** Wound healing assay after transient silencing and overexpression of circRREB1. (**C)** Statistical analysis of the wound healing assay results. (**D**) The CCK-8 assay was performed to assess cell viability following the simultaneous silenced of ALYREF and overexpression of circRREB1. (**E**) The transwell assay was conducted to assess the cell migration following silenced of ALYREF and overexpression of circRREB1. (**F**) Statistical analysis of the transwell assay results. (**G)** Fluorescence of stable circRREB1-silenced cell lines. (**H)** qPCR was used to assess the efficiency of stable silencing of circRREB1 in cell lines. (**I)** Statistical analysis of immunohistochemical detection of Ki67 expression in tumours from nude mice. (**J)** Statistical analysis of immunohistochemical detection of cyclin D1 expression in tumours from nude mice. (**K)** Statistical analysis of the results of the immunohistochemical detection of BCL2 expression in tumours from nude mice. **(L)** Statistical analysis of the results of the immunohistochemical detection of RhoA expression in tumours from nude mice.

Supplementary Fig. S3

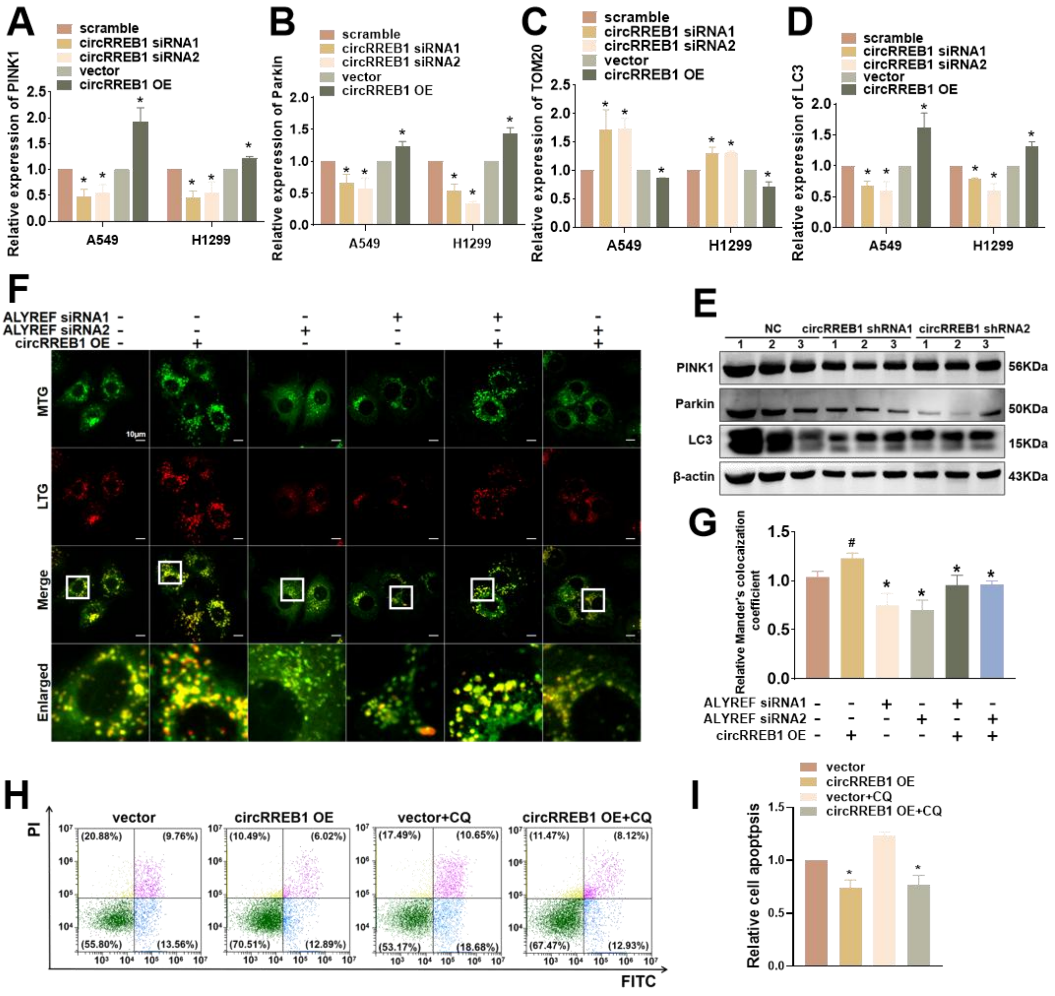

Supplementary Fig. S3 circRREB1 regulation of mitophagy. (**A)** Statistical analysis of PINK1 protein expression following transient silencing and overexpression of circRREB1. (**B)** Statistical analysis of Parkin protein expression following transient silencing and overexpression of circRREB1. (**C)** Statistical analysis of TOM20 protein expression following transient silencing and overexpression of circRREB1. (**D)** Statistical analysis of LC3 protein expression following transient silencing and overexpression of circRREB1. (**E**) WB was performed to detect mitophagy-related proteins extracted from tumour tissues of nude mice. (**F**) Mitochondrial-lysosomal colocalization was detected following ALYREF silencing and circRREB1 overexpression. Scale bar, 10 μm. MTG: MitoTracker Green; LTR: LysoTracker Red. (**G**) Statistical analysis of the mitochondrial-lysosomal co-localization assay results for F. (**H)** Flow cytometry detection of apoptosis in cells overexpressing circRREB1 and treated with chloroquine. (**I)** Statistical analysis of apoptosis detected by flow cytometry after overexpression of circRREB1 with the addition of chloroquine.

Supplementary Fig. S4

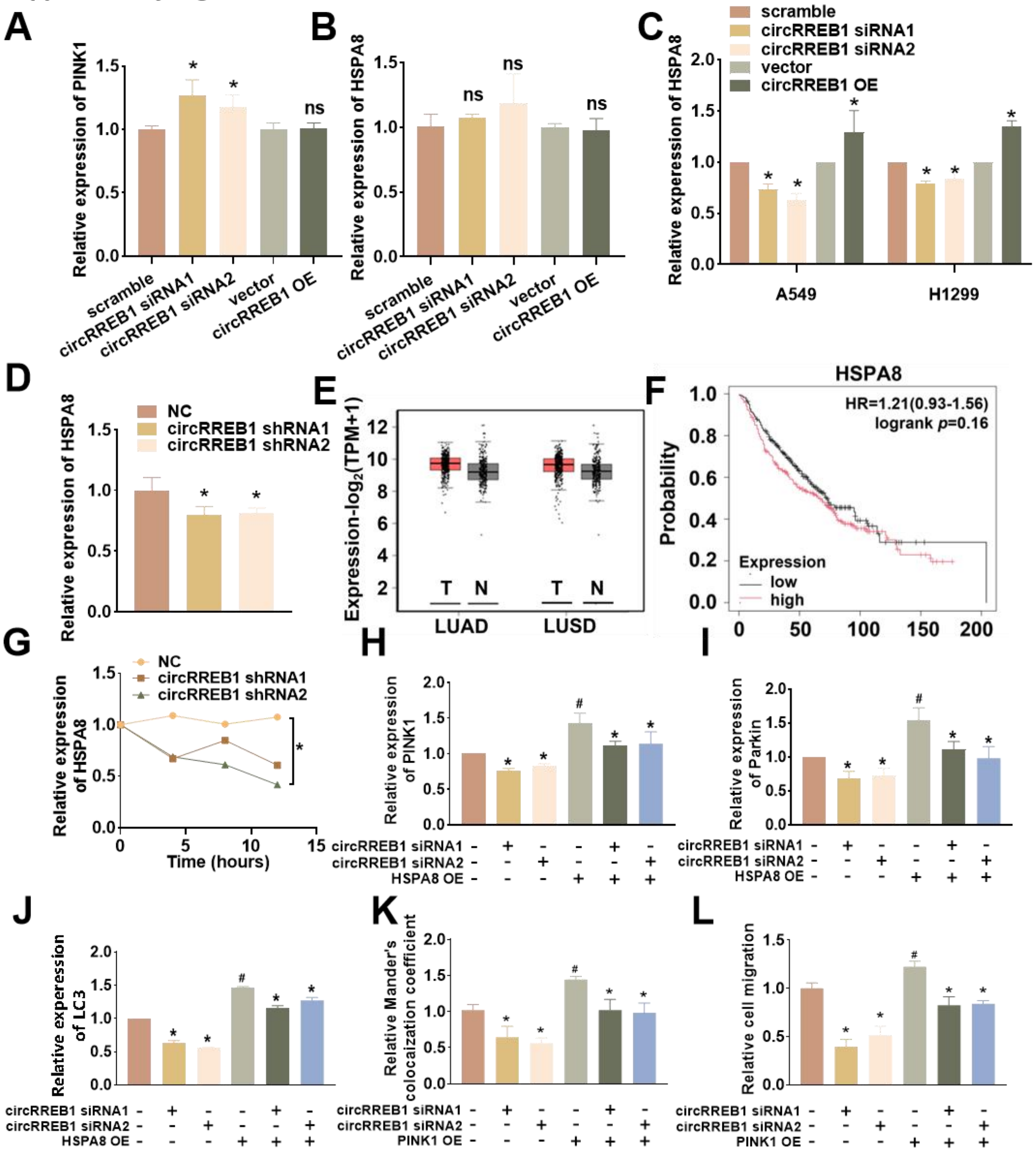

Supplementary Fig. S4 Analysis of circRREB1 regulation of HSPA8 and statistical analysis of rescue experiments. (A) qPCR was used to measure PINK1 mRNA expression levels following transient silencing and overexpression of circRREB1. (**B)** qPCR was used to measure HSPA8 mRNA expression levels following transient silencing and overexpression of circRREB1. (**C)** Statistical analysis of HSPA8 protein expression following transient silencing and overexpression of circRREB1. (**D)** Statistical analysis of the immunohistochemical detection of HSPA8 expression in tumours from nude mice. (**E)** Expression levels of HSPA8 in lung cancer and adjacent non-cancerous tissues. (N, nontumorous tissue; T, tumorous tissue). (**F)** Survival curve analysis for patients grouped according to HSPA8 expression. (**G)** Statistical analysis of HSPA8 expression levels after treatment with CHX (50 μg/ml). (**H)** Expression of the PINK1 protein in the rescue experiment. (**I)** Expression of the Parkin protein in the rescue experiment. **(J)** Expression of the LC3 protein in the rescue experiment. (**K**) Statistical analysis of mitochondrial-lysosomal colocalization results. (**L)** Statistical analysis of the transwell migration assay results.
